# Assessment of developmental rate of mouse embryos yielded from in vitro fertilization of the oocyte with treatment of melatonin and vitamin C simultaneously

**DOI:** 10.1186/s12905-023-02673-w

**Published:** 2023-10-04

**Authors:** Shadan Navid, Zahra Saadatian, Ali Talebi

**Affiliations:** 1 Department of Anatomy, Faculty of Medicine, Social Determinants of Health Research Center, Gonabad University of Medical Science, Gonabad, Iran; 2 Department of Physiology, Faculty of Medicine, Infectious Diseases Research Center, Gonabad University of Medical Sciences, Gonabad, Iran; 3 School of Medicine, Shahroud University of Medical Sciences, Shahroud, Iran; 4 Sexual Health and Fertility Research Center , Shahroud University of Medical Sciences, Shahroud, Iran

**Keywords:** In vitro fertilization, Melatonin, Vitamin C, Cumulus-oocyte complexes, Development

## Abstract

**Background:**

In recent decades, in vitro fertilization *(*IVF*)* has been widely used as a method of assisted reproductive technology (ART) to improve fertility in individuals. To be more successful in this laboratory method, we used the presence of two common types of antioxidants (melatonin and vitamin C) simultaneously and exclusively in IVF medium.

**Methods:**

The cumulus-oocyte complexes (COCs) were obtained from Gonadotropin-releasing hormone (GnRH) and Human Chorionic Gonadotropin (HMG) -stimulated mice. Subsequently, metaphase II (MII) oocytes were fertilized in vitro. In the experiment, the IVF medium was randomly divided into two equal groups: The control group did not receive any antioxidants. In the treatment group, 100 µM melatonin and 5 mM vitamin C were added to the IVF medium. Finally, oocytes and putative embryos transferred into developmental medium and cultured 120 h after IVF to the blastocyst stage. After and before IVF, oocytes and putative embryos were stained with dichlorodihydrofluorescein diacetate (DCFDA) and the H2O2 level was measured with an inverted fluorescence microscope using ImageJ software. At the end of the fifth day after IVF, the expression of Bax and B cell lymphoma 2 (Bcl2) was evaluated using real-time PCR.

**Results:**

The levels of reactive oxygen species (ROS) in oocytes and putative embryos observed in the treatment group demonstrated a significant reduce compared to the control group (p ≤ 0.01. (.Furthermore, the number of embryos in the blastocycte stage(P < 0.05), the expression level of the Bcl2 (P < 0.05) gene, the Bax unlike gene, significantly increased compared with the control group.

**Conclusion:**

We conclude that the presence of melatonin and vitamin C antioxidants simultaneously and exclusively in the IVF medium leads to a reduction in ROS and ,as a result, improves the growth of the embryo up to the blastocyst stage.

## Introduction

Infertility is defined as the inability of a couple to conceive after 12 months of regular and unprotected intercourse. According to statistics, this condition affects 8 to 12% of couples worldwide [1]. In vitro embryo production (IVEP) poses many challenges, such as lower quality and less programmable in vitro fertilized oocytes compared to those in vivo oocyte conditions [2].

The complete maturation of an oocyte depends on its nuclear and cytoplasmic changes. Defects in these mechanisms can reduce the chances of successful reproduction [3]. The composition of follicular fluid plays a very important role in the growth and development of oocytes and embryos [4].

Presence of oxygen species (ROS) in the culture medium is one of the primary reasons for impaired oocyte growth and development after in vitro fertilization [5]. The function of oxidative stress (OS) is to increase the induction of apoptosis in granulosa cells, which leads to the oocyte lacking access to nutrients and growth [6]. To eliminate oxidative stress in culture medium, various types of antioxidants have been used to date.

Melatonin (N-acetyl-5-methoxytryptamine) is a circadian hormone that plays a crucial role in regulating the body’s activities, particularly reproductive activity. As an antioxidant, melatonin improves oocyte function, reduces apoptosis, and modifies chromosomes and spindle arrangement [2, 7–10]. In a study, melatonin was loaded onto nanostructure lipid carriers (Mel-NLCs), and the effect of oocyte and embryo development in the IVF medium was investigated. The results were positive [11]. Women who exhibit a low fertilization rate during oocyte retrieval for in vitro fertilization and embryo transfer may experience an inverse correlation between the level of 8-hydroxy-2′-deoxyguanosine (8-OHdG) in their follicular fluid, a marker of oxidative stress, and the level of melatonin, a hormone known for its antioxidant properties. The administration of melatonin treatment has been shown to decrease the concentration of 8-OHdG in the follicular fluid, thereby potentially improving the fertilization outcome [12]. Many articles have published the therapeutic effects of melatonin in the treatment of oocyte and embryo development in vitro [13–22]. Vitamin C is another antioxidant used to improve cell culture and development and has anti-aging effects [23, 24].

Vitamin C has been shown to improve in vitro maturation (IVM) of bovine oocytes and increase the number of blastocysts [25]. Several studies have reported similar findings [26–28].

In this study, the co-occurrence of melatonin and vitamin C solely within the IVF medium may represent a novel approach to enhancing the processes of fertilization and embryo culture.

## Methods

### Animals

The naval medical research institute (NMRI) mice (male and female) were used. They had free access to water and food. All steps of working with animals were performed in accordance with the ethics regulations in research at Shahroud University of Medical Sciences. This study design is experimental.

### IVF, embryo culture

Female NMRI mice (4 to 6 weeks) (5 mice at a time in each group (received 5 IU Gonadotropin-releasing hormone (GnRH; Organon, Holland) by intraperitoneal(IP) injection. After 46–48 h, 5 IU Human Chorionic Gonadotropin (HCG; LG Life, Sciences Korea) was injected to induce superovulation.

14–16 h after induction of ovulation, by killing female mice by cervical dislocation, cumulus-oocyte complexes (COCs) were collected from the fallopian tube by squeezing with a G 29 needle into the swollen area, and into small droplets of IVF culture medium (G-IVF,

Vitrolife. Gothenburg, Sweden) covered with mineral oil(Sigma) and then transferred to a humidified incubator at 37 ° C and 5% CO2.

Under inverted microscopy, the collected oocytes were evaluated. Healthy metaphase II (MII) oocytes have a clear cytoplasm, and the polar body (PB) in the yolk period space. These oocytes were transferred to IVF medium for further experiments. Degenerated oocytes whose cytoplasm was compressed. Degenerated oocytes were excluded from the study. Immature oocytes with germinal vesicle (GV) and metaphase I(MI) oocytes lacking GV and PB were also excluded.

Capacitated sperms were obtained from the cauda epididymis of adult male mice after incubation for 1 h in droplets containing IVF medium. The concentration of sperm was determined using a Makler counting chamber, and approximately 1 to 2 million motile sperm per ml that were added to each COC and allowed to fertilize for 4 h.

The IVF medium was randomly divided into two equal groups: The control group, it does not receive any antioxidants. In the treatment group, 100 µM melatonin (Sigma) [29] and 200 µM vitamin C(Sigma) [30],as antioxidants, were added to the IVF medium.

Four hours after IVF, putative embryos were evaluated for two pronucleus (2PN) formation (the presence of separate or fused male and female peronuclei) under an inverted microscope and transferred to fresh development droplets. These drops contain G1 and G2 media in the.

EmbryoScope™ (Vitrolife, Gothenburg, Sweden). The embryos were then monitored daily to be evaluated for developmental and fragmentation assessment, which continued until 120 h after IVF and the rate of embryo maturation from the two-cell stage to the blastocyst was evaluated under an inverted microscope [29, 31].

### Intracellular ROS measurement

The intracellular H2O2 content of oocytes and putative embryos was measured before and after incubation with sperm in IVF medium. Oocytes and putative embryos were washed 3 times in phosphate-buffered saline (PBS) and then incubated in PBS supplemented with 10 μm DCFDA (10 ml, Sigma, Life Technologies C6827) for 15 min at 37 °C and subsequently slowly washed with PBS. The stained oocytes or putative embryos were transferred into a droplet on a glass slide and observed by an inverted fluorescence microscope with excitation wavelength at 488 nm. Finally, the pixel intensity within recorded fluorescent images was analyzed using ImageJ software [32].

### Real-time PCR

The BAX gene, belonging to the Bcl-2 gene family, encodes a protein that serves as an activator of apoptosis. Its role in the regulation of programmed cell death, a biological process that takes place in multicellular organisms, is well-established. The gene B-cell CLL/lymphoma 2 (BCL2) is responsible for encoding a protein that serves as an integral outer mitochondrial membrane protein with the capacity to impede the apoptotic death of a cell. The BCL2 family members are capable of forming hetero- or homodimers and serve as either anti- or pro-apoptotic regulators that are involved in a diverse range of cellular activities.

Embryos were collected in the blastocyst stage, and a real-time technique was used to evaluate the expression levels of apoptosis regulator (Bax, proapoptotic) and B cell lymphoma 2 (Bcl2, antiapoptotic). All RNA from blastocysts was isolated according to the manufacturer’s instructions and using theTRIzol reagent (Ready Mini Kit, Qiagen) protocol. The step one real time PCR system (Applied Biosystems, Waltham, MA) was employed in our study. All samples were normalized using the comparative CT method (ΔΔCT). Glyceraldehyde-3-phosphate dehydrogenase (GAPDH) housekeeping gene was used in this method. Primers were designed by allele ID software. The sequences of the primers are shown in Table 1 [33].

**Table 1:** The primers sequence *Bax, Bcl2, GAPDH* of genes

Gene name	Sequence	Product size (bp)	annealing temp
***Bcl2***	For: 5’- GCAAACTGGTGCTCAAGG − 3’ Rev: 5’- CAGCCACAAAGATGGTCA − 3’	**183**	**56.63**
***Bax***	For: 5’- GAGTGGGATACTGGAGATGAAG − 3’ Rev: 5’- TGGTAGCGACGAGAGAAGTCC − 3’	**233**	**57.4**
***GAPDH***	For: 5’- AAGTTCAACGGCACAGTCAAGG − 3’ Rev: 5’- CATACTCAGCACCAGCATCACC − 3’	**121**	**61.58**

### Statistical analysis

All data were communicated as the mean – standard deviation. All statistical analysis of data were performed using one-way analyses of variance (ANOVA) followed by Tukey’s post hoc test.

p ≤ 0.05 was considered statistically significant.

## Results

### Morphology of different stages of IVF to blastocyst embryo

**Fig. 1:**
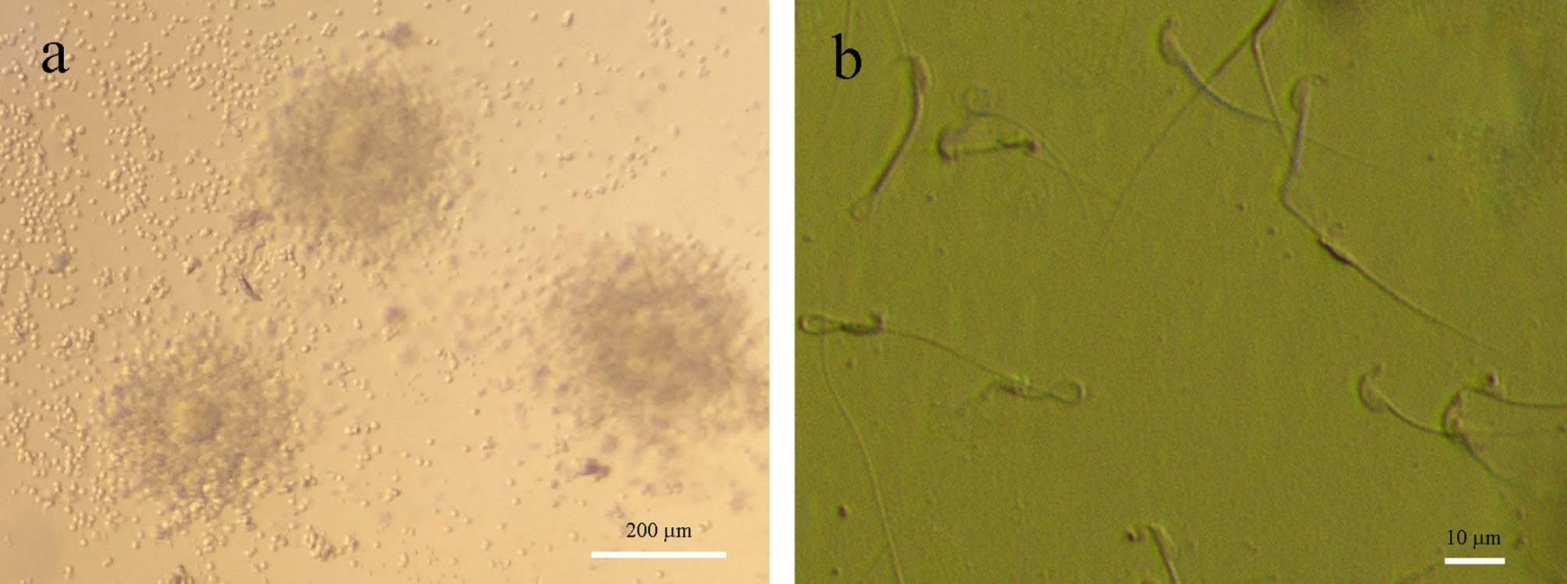
Inverted microscopy morphology of COCs includes MII oocytes with normal cytoplasm enclosed by Corona radiate (**a**) and Capacitated sperms (**b**) mice. (Inverted microscope, scale bar = 40 μm)

The collection of COCs included oocytes enclosed by Corona radiata cells that were healthy in quality MII oocytes with normal cytoplasm and appropriate size, and capacitated sperm were examined using an inverted microscope(Fig. 1).

**Fig. 2:**
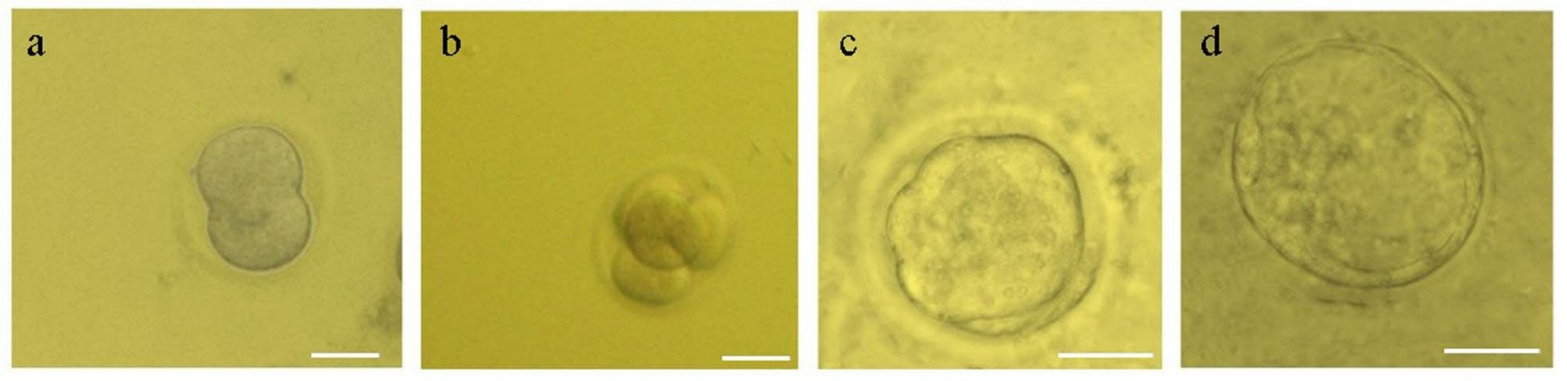
The image shows the growth and cell divisions of the embryo in different stages. 2-cell embryo (**a**), eight -cell embryo (**b**), morula (**c**) and blastocyst (**d**) embryos. we have used 5 mice per group and per each type of experiment least 3 replicate were applied. Each embryo culture repeated least 5 to 6 times

As shown in Fig. 2, the images show the growth and cell divisions of the embryo in different stages.

### Embryo development rate in the control group and treatment group (melatonin and vitamin C)

The existence of antioxidants exclusively to in the IVF medium increases the percentage of fertilization and embryonic growth at all stages of development, including 2-cell embryo, morula, and blastocyst embryos. However, it significantly increased the development rate (% ± SD) of embryos during fertilization (77.5 ± 3.5) and blastocyst formation (32.5 ± 1.825).

**Fig. 3:**
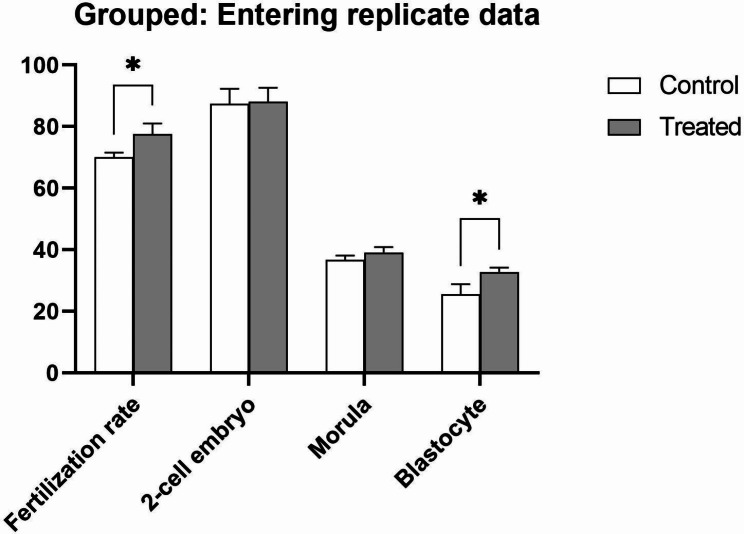
The number of oocytes and embryos in the development rate stages of 2-PN, 2-cells, morula, blastocyst. Data show means ± SD; *p ≤ 0.05

These results show that the presence of antioxidants solely in the IVF medium had a significant effect on both the fertilization rate and blastocyst formation rate (p ≥ 0.05) (Fig. 3). According to the values in Table 2.

**Table 2:** The number of MII oocytes and development rate of mouse embryos in-vitro

Groups	Numbr of MII oocytes	2-PN embryo development rate (% ± SD)	2-cell embryo development rate (% ± SD)	Morula embryo development rate (% ± SD)	Blastocyst embryo development rate (% ± SD)
Control	258	70.06 ± 1.48	87.4 ± 4.91	36.73 ± 1.35	25.56 ± 3.21
Antioxidant supplemented	273	77.5 ± 3.58 *	88 ± 4.66	39.13 ± 1.75	32.5 ± 1.825 *

### Measurement of intracellular ROS production of oocytes and putative embryos before and after incubation with sperm in IVF medium

**Fig. 4:**
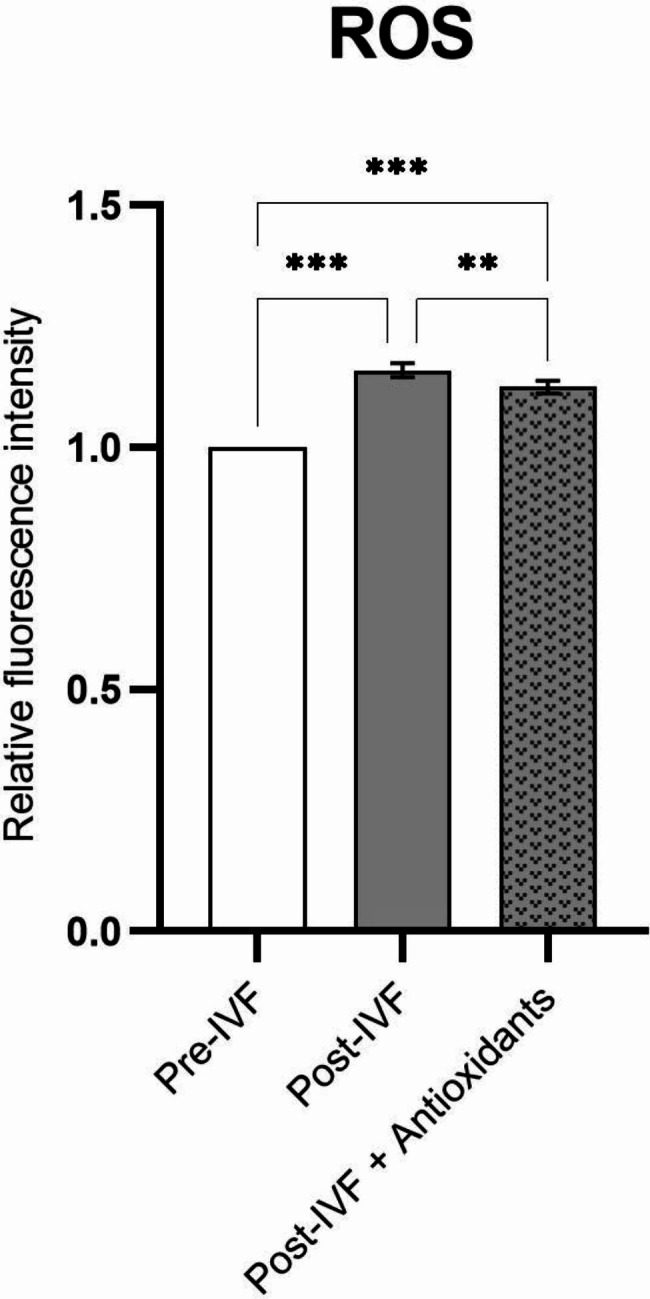
Inverted fluorescence microscope for measurement of intracellular ROS production of oocytes and putative embryos before and after incubation with sperms in IVF medium in different groups. Data show mean ± SD; **P ≤ 0.01, ***P ≤ 0.001

Measurement of intracellular ROS production, oocytes and putative embryos were measured before and after incubation with sperms in IVF medium in )both the control group and antioxidant group (was carried out by an inverted fluorescence microscope with excitation wavelength at 488 nm. The pixel intensity within recorded fluorescent images was then analyzed using ImageJ software. ROS production in the post-IVF (oocytes and putative embryosب) group was significantly higher compared with the pre-IVF (oocytes) group(p ≤ 0.001) and post-IVF + antioxidant (oocytes and putative embryos with antioxidant) group(p ≤ 0.001). These results suggest that supplementation of IVF medium with melatonin and vitamin C can greatly reduce intracellular ROS production and improve embryonic development (Fig. 4).

### Evaluation of apoptotic changes in blastocyst embryos using the presence of antioxidants in the fertilization stage

**Fig. 5:**
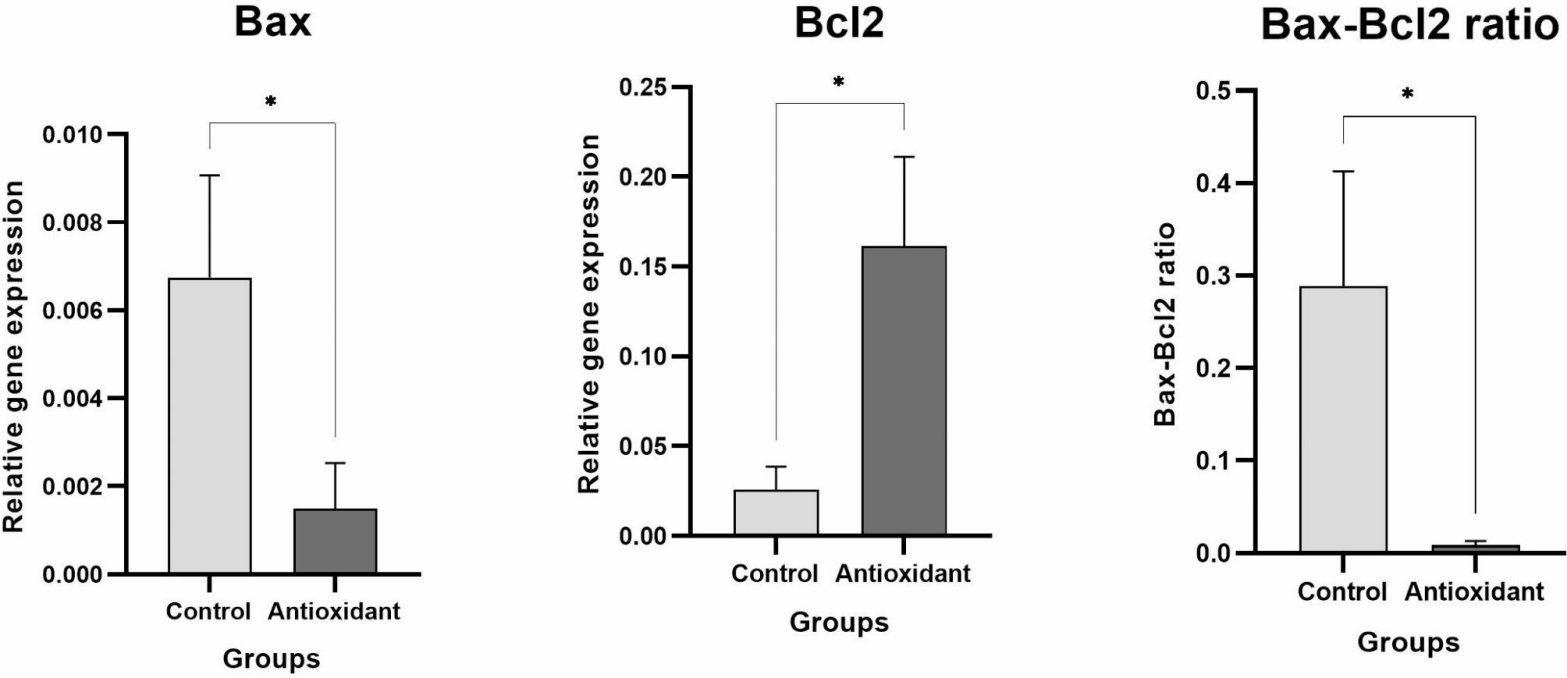
Changes in the expression of Bax and Bcl2 genes in control and Antioxidant groups, 5 days after IVF. Bax expression and Bcl2 genes were significantly different. In addition, the expression the ratio of Bax - Bcl2 genes in the control group is significantly higher than the antioxidant group. Data show means ± SD; *p ≤ 0.05

To further investigate apoptotic changes in blastocyst embryos after the use of antioxidants in the fertilization medium, 5 days after IVF, changes in the expression of Bax and Bcl2 genes and the ratio of Bax - Bcl2 genes involved in apoptosis were examined by real-time PCR. As shown in Fig. 5, in the control and antioxidant groups, Bax expression (proapoptosis) and Bcl2 genes (antiapoptosis) were significantly different (p ≤ 0.05). In addition, the expression ratio of the Bax - Bcl2 genes in the control group is significantly higher than that in the antioxidant group (p ≤ 0.05).

In many studies, the expression level and ratio of expression of the apoptotic gene, box gene, and anti-apoptotic gene, Bcl2 gene, have been utilized to demonstrate the enhancement of cell culture and development.

This graph clearly shows that the presence of antioxidants exclusively in the of fertilization medium led to a reduction in apoptosis and improved embryo growth development prior to the implantation stage(Fig. 5).

## Discussion

In the present study, we used a combination of two antioxidants, melatonin and vitamin C, exclusively in the IVF medium of mice before and after incubation. We measured the production of reactive oxygen species (ROS) in oocytes and putative embryos before and after incubation with sperm in IVF medium, as well as the expression levels of Bax and Bcl2 genes. Our results suggest that the presence of antioxidants exclusively in the IVF medium can improve the development and culture of embryos.

Vitamin C has long been known to play an important role in improving health and reducing the aging process. Therefore, we used this vitamin as one of the antioxidants in the IVF medium [34, 35]. Several studies have investigated the effect of ascorbic acid (vitamin C) on the development of embryos in vitro and obtained results similar to our research [36–39].

In another study, it was found that vitamin C plays an important role in correcting DNA demethylation by modulating ten-eleven translocation (TET) dioxygenases that cause DNA demethylation. Therefore, incubating vitamin C in the embryo development medium can be effective in correcting epigenetic errors [40].

another part of Yeon et al’s research, α-tocopherol was used as an antioxidant both alone and as a supplement with vitamin C. When added alone to the developing medium, α-tocopherol had a similar effect to vitamin C. However, when used as a supplement with vitamin C, it had no effect on improving the quality of blastocysts. Alpha-tocopherol or L-ascorbic acid reduced the proportion of apoptotic cells in blastocysts, but their combination led to the same apoptosis rate as the control group. In our research, we also used vitamin C and melatonin as antioxidants at the same time, and the results showed that the presence of two antioxidants simultaneously in the IVF medium had a positive role in improving the quality of embryos and reducing free radicals. Therefore, this part of Yeon et al’s research contradicts our findings [41].

In a similar study, Husamaldeen et al. investigated the effect of the antioxidant cysteamine with vitamin C on in vitro fertilization in cows. The results of their research showed that the presence of vitamin C in the culture medium improves the fertilization of cow oocytes. However, the presence of both cysteamine and ascorbic acid (vitamin C) antioxidants did not improve the fertilization of cow oocytes [30]. Since the simultaneous use of vitamin C and cysteamine as antioxidants in the fertilization of cow oocytes did not have a positive effect on improving the fertilization of oocytes, their results contradict ours.

Similar to our research, vitamin C has been proven to be an effective antioxidant in improving cultivation [30, 41].

Castillo-Martín et al. conducted a study in which porcine blastocysts were placed in vitro culture medium and/or vitrification–warming media supplemented with vitamin C. The researchers then analyzed embryo quality in terms of total cell count (TCN), DNA fragmentation, peroxide levels, and the relative transcript abundance of BCL-associated protein X (BAX), BCL2-like 1 (BCL2L1), POU class 5 homeobox 1 (POU5F1), and heat shock protein 70 (HSPA1A) (42). In our research, we also used the Bax and Bcl2 genes to evaluate the viability and development of blastocysts, similar to Castillo-Martín et al.‘s research.

Boldura et al. used vitamin C and rosmarinic acid to improve the development and growth of sow oocytes in the culture medium for 44 h. At the end of the study, Ptx3 genes and apoptosis regulators p53, Bax, and BCL-2 were analyzed. The results showed that the presence of vitamin C plays a positive role in increasing the quality of embryos, similar to our research [43].

Melatonin is a useful antioxidant in culture medium that plays an effective role in reducing free radicals. It increases the expression of ATPase 6, BMP-1GDF-9, SOD-1, Gpx-4, and Bcl-2, which are vital genes in oocyte maturation and embryo development. Additionally, it decreases the expression of caspase 3, [44] which confirms the need for antioxidants to neutralize free radicals produced in vitro. Melatonin has been used as an effective factor in the culture medium in many studies as a free radical reducer. It helps the growth and development of cells in vitro [45–47] and has been investigated for its positive effect on IVF quality and embryo growth and development [12, 14, 29, 48–50].

In other research, melatonin was used as a supplement with mouse two-cell embryos in cryogenic medium and vitrified by cryotop. The results of the research showed that a high percentage of embryos that were exposed to melatonin reached the blastocyst stage [22]. Unlike our research, this research used melatonin as an antioxidant in vitrification cryopreservation.

Similar to our results, Bahadori et al. [2013] also showed the positive effect of melatonin on the growth and development of oocytes and embryos [29].

Our research has some limitations. For instance, the use of antioxidants as a supplement in the fertilization medium increases the possibility of contamination. Additionally, fertilization and subsequent embryo culture are difficult processes, and using different supplements can make this process more difficult and costly. However, our research stands out from other studies in this [40, 41] because we used antioxidants as a supplement only in the fertilization medium, which led to a reduction in costs.

Our findings indicate that adding 100 µM melatonin and 200 µM vitamin C as antioxidants reduces intracellular ROS production of oocytes and improves the quality and development of embryos up to the blastocyst stage, but only in the IVF medium.

## Data Availability

The datasets generated and analyzed during the current study are not publicly available due to its proprietary nature or ethical concerns, supporting data cannot be made openly available but are available from the corresponding author on reasonable request.“ No anesthesia or euthanasia was used in our study.
